# Influence of Lavender Essential Oil on the Physical and Antibacterial Properties of Chitosan Sponge for Hemostatic Applications

**DOI:** 10.3390/ijms242216312

**Published:** 2023-11-14

**Authors:** Daniela Gheorghiță, Iulian Antoniac, Horațiu Moldovan, Aurora Antoniac, Elena Grosu, Ludmila Motelica, Anton Ficai, Ovidiu Oprea, Eugeniu Vasile, Lia Mara Dițu, Anca Daniela Raiciu

**Affiliations:** 1 Faculty of Material Science and Engineering, National University of Science and Technology Politehnica Bucharest, 313 Splaiul Independentei, 060042 Bucharest, Romania; daniela.mgm8@gmail.com (D.G.); antoniac.iulian@gmail.com (I.A.); elena_grosu@yahoo.com (E.G.); 2 Academy of Romanian Scientists, 54 Splaiul Independentei, 050094 Bucharest, Romania; anton_ficai81@yahoo.com (A.F.); ovidiu73@yahoo.com (O.O.); 3 Faculty of Medicine, Carol Davila University of Medicine and Pharmacy, 050474 Bucharest, Romania; 4 Department of Cardiovascular Surgery, Emergency Clinical Hospital Bucharest, 014461 Bucharest, Romania; 5 National Research Center for Micro and Nanomaterials, National University of Science and Technology Politehnica Bucharest, 060042 Bucharest, Romania; motelica_ludmila@yahoo.com; 6 National Research Center for Food Safety, National University of Science and Technology Politehnica Bucharest, Splaiul Independentei 313, 060042 Bucharest, Romania; 7 Faculty of Chemical Engineering and Biotechnologies, National University of Science and Technology Politehnica Bucharest, 1-7 Polizu St., 011061 Bucharest, Romania; 8 Department of Oxide Materials Science and Engineering, National University of Science and Technology Politehnica Bucharest, 1–7 Gh. Polizu, 060042 Bucharest, Romania; eugeniu.vasile@upb.ro; 9 Botanic and Microbiology Department, Faculty of Biology, University of Bucharest, 3, Aleea Portocalelor, 17 District 5, Grădina Botanică, 030018 București, Romania; lia_mara_d@yahoo.com; 10 Faculty of Pharmacy, Titu Maiorescu University, 22 Dambovnicului Street, 040441 Bucharest, Romania; daniela_raiciu@yahoo.com; 11 S.C. Hofigal Import Export S.A., 2 Intrarea Serelor Street, 042124 Bucharest, Romania

**Keywords:** chitosan, lavender essential oil, hemostatic applications, silver nanoparticles, antibacterial activity

## Abstract

Uncontrollable bleeding continues to stand as the primary cause of fatalities globally following surgical procedures, traumatic incidents, disasters, and combat scenarios. The swift and efficient management of bleeding through the application of hemostatic agents has the potential to significantly reduce associated mortality rates. One significant drawback of currently available hemostatic products is their susceptibility to bacterial infections at the bleeding site. As this is a prevalent issue that can potentially delay or compromise the healing process, there is an urgent demand for hemostatic agents with antibacterial properties to enhance survival rates. To mitigate the risk of infection at the site of a lesion, we propose an alternative solution in the form of a chitosan-based sponge and antimicrobial agents such as silver nanoparticles (AgNPs) and lavender essential oil (LEO). The aim of this work is to provide a new type of hemostatic sponge with an antibacterial barrier against a wide range of Gram-positive and Gram-negative microorganisms: *Staphylococcus epidermidis* 2018 and *Enterococcus faecalis* VRE 2566 (Gram-positive strains) and *Klebsiella pneumoniae* ATCC 10031 and *Escherichia coli* ATCC 35218 (Gram-negative strains).

## 1. Introduction

At times, individuals may find themselves in traumatic situations involving injuries that lead to either internal or external bleeding, which is often agonizing. The causes of injuries are multiple and happen during unpleasant and unexpectable events such as surgical interventions, accidents, lacerations, punctures, diabetic wounds, and accidents during combat on the battlefield. When the blood loss is up to 15% of the entire volume, the bleeding is called a hemorrhage [1,2,3,4,5,6].

Uncontrolled bleeding caused by a traumatic injury can give rise to numerous complications, including hypothermia, coagulopathy, reduced blood pressure, acidosis, bacterial infection, and multiple organ failure. During surgical procedures, excessive bleeding disrupts the patient’s hemodynamics, prolongs the duration of the operation, and elevates the risk of infection from transfusions. These complications significantly contribute to the escalation of healthcare expenses and pose a life-threatening danger to the patient [7,8,9,10]. Therefore, to mitigate the risk of complications and reduce the chances of morbidity and mortality associated with a hemorrhage, it is crucial to use an effective hemostatic agent that can promptly achieve bleeding control [11,12,13].

The process of wound healing occurs through four intricate phases: hemostasis, inflammation, proliferation, and remodeling. Hemostasis represents the first phase in the wound healing process and is a critical process that involves multiple interconnected steps to prevent and stop blood loss resulting from the disruption of blood vessel walls. Hemostasis is vital for survival, especially in cases of severe injuries, when an important hemorrhage threatens the patient’s life [14,15,16,17].

While an excessive hemorrhage remains the primary cause of death, accounting for 80% of early trauma-related fatalities, it is important to note that deaths occurring within five days of the injury are primarily attributed to infections [18,19]. Also, surgical site infections are recognized as one of the leading causes of morbidity and mortality in various surgical procedures [20,21,22,23]. Considering that infection is a commonly encountered issue that can impede or delay the healing process, there is a pressing demand for hemostatic agents with antibacterial properties. The availability of such agents would be vital to improving survival rates [24,25,26].

Due to the absence of protective barriers in the body, along with the moist and nutrient-rich environment of the wound, favorable conditions are created for bacterial growth. This, in turn, leads to infections that trigger the systemic immune response and hinder vital processes involved in wound healing [27,28].

In recent years, there has been a notable emphasis on the development of novel materials that integrate hemostatic and antibacterial properties. This focus arose from the frequent occurrence of both bleeding and infection in traumatic wounds, as well as the escalating incidence of infections caused by pathogenic strains and the concerning rise of antibiotic resistance [29]. Therefore, there is a significant interest in promoting hemostasis, preventing wound infections, and enhancing the wound healing process, when it comes to hemostatic agents. These aspects should be given serious consideration in the development and engineering of hemostatic materials [11,30,31]. 

An effective hemostatic material should possess several key characteristics. It should be easy and convenient to apply, ideally adhering well to the bleeding surface. Additionally, it should induce rapid blood clotting, securely hold the blood clot in place to prevent delayed bleeding, and preferably create a reliable seal for the damaged blood vessel. Furthermore, it would be advantageous if the hemostat could also seal the wound, thereby preventing contamination and infections. Importantly, it should not interfere with the natural wound healing process [2,32,33,34,35].

As an immediate first aid application, topical hemostatic sponges are very useful for stopping bleeding at the site of lesion. Many studies on different materials were conducted using some categories of devices based on natural and synthetic materials to stop bleeding. Shortly, we can enumerate the following: active or adhesive hemostats (based on fibrin, thrombin, gelatin combined with thrombin, gelatins, collagen, oxidized cellulose, and polysaccharides), synthetic hemostatic agents (cyanoacrylates, hydrogel based on polyethylene glycol, albumin cross-linked with glutaraldehyde, and synthetic molecules), external hemostatic dressings (based on fibrinogen, zeolites, clay (kaolin and smectite), chitin, chitosan, polysaccharides, and polyelectrolytes) to be used on the battlefield in order to stop severe bleedings in precarious conditions [36,37,38,39,40].

The hemostatic mechanism is produced by the adhesion, aggregation, and activation of platelets [24,27]. For example, devices based on chitosan, when applied to wounds, promote hemostasis by plasma absorption, the aggregation of plasma proteins, and platelet adhesion [41,42]. When applied to an injured site, dressings based on chitosan create an anionic environment and, therefore, induce blood clotting. Also, chitosan generates an antibacterial zone that favors healing [43,44,45,46,47,48,49]. 

Some essential oils were the subject of numerous analyses for finding a solution to improve the antimicrobial activity of different products that come in contact with the human body. For example, essential oil extracted from the Chinese herb Qiai was tested by citomembrane permeability, the leakage of the cell constituent, and electron microscopy and demonstrated antimicrobial properties against Gram-positive and Gram-negative bacteria [50]. Some studies performed on essential oils from *Asteraceae* species demonstrate properties of clotting or fibrinogenolysis that allow for their use in the treatment of snakebites [51]. Moreover, some models of hemostatic devices were developed, such as a triple-protective aromatic patch, that contain two polymeric layers (a permeable backing and a drug or essential oil loaded in a pressure-sensitive adhesive (PSA) and two release liners, one for each layer, upper and lower) [52].

Lavender stands as one of the world’s most frequently utilized aromatic plants, with its extracts and essential oils having a longstanding tradition of being employed to address conditions like epilepsy and migraines. Lavender inspired and propelled the cosmetics industry and, more recently, the food supplements industry [53,54]. Inspired by the healing properties of lavender, known and successfully exploited in the past centuries, researchers are currently studying the expansion of the fields of use, such as in dentistry and surgery. Due to their content in linalool, linalyl acetate, 8-cineole-ocimene, terpinen-4-ol, camphor, and others [55,56], the essential oils of lavender possess antimicrobial properties, especially against Gram-positive and Gram-negative microorganisms and fungi. Among them, *Candida albicans*, *Staphyloccocus aureus*, *Aspergillus nidulans*, *Trichophyton mentagrophytes*, and *Escherichia coli* can be enumerated. Many studies revealed the increasing antimicrobial activity of some antibiotic drugs, by the use of the essential oils of lavender. Some special properties of the essential oils of lavender, such as being anxiolytic during surgery, anti-inflammatory, and antimicrobial, were revealed in several studies [57,58,59,60,61,62,63]. 

Metallic and metallic oxide nanoparticles, including ZnO, copper, silver, gold, and platinum, are commonly known to possess bactericidal properties [64,65,66]. Among these inorganic antibacterial agents, silver ions and compounds were extensively investigated and studied [67,68,69,70]. Silver nanoparticles (AgNPs) possess the capability to interact with a diverse range of microorganisms, including mature bacterial biofilms. Their antimicrobial activity was extensively documented for over 650 microorganisms, encompassing bacteria, fungi, and even viruses [71,72,73,74,75,76]. Consequently, they have the potential to be employed as broad-spectrum antimicrobials capable of targeting a wide range of pathogens [77,78,79].

Silver nanoparticles (AgNPs) adhere to bacterial surfaces through Coulomb attraction forces, enabling them to penetrate the cell wall [80,81]. Once inside the bacterial cell, AgNPs bind to intracellular macromolecules such as oxidative metabolic enzymes and DNA. This interaction causes structural alterations in the bacterial DNA and disrupts bacterial metabolism. Consequently, incorporating AgNPs into chitosan materials enhances antibacterial properties [82,83,84,85,86].

To mitigate the risk of infection at the site of a lesion, we propose an alternative solution in the form of a chitosan-based sponge and antimicrobial agents such as silver nanoparticles (AgNPs) and lavender essential oil (LEO). The aim of this work is to provide a new type of hemostatic sponge with an antibacterial barrier against a wide range of Gram-positive and Gram-negative microorganisms: *Staphylococcus epidermidis* 2018 and *Enterococcus faecalis* VRE 2566 (Gram-positive strains) and *Klebsiella pneumoniae* ATCC 10031 and *Escherichia coli* ATCC 35218 (Gram-negative strains).

## 2. Results and Discussion

### 2.1. ATR-FTIR

**Figure 1:**
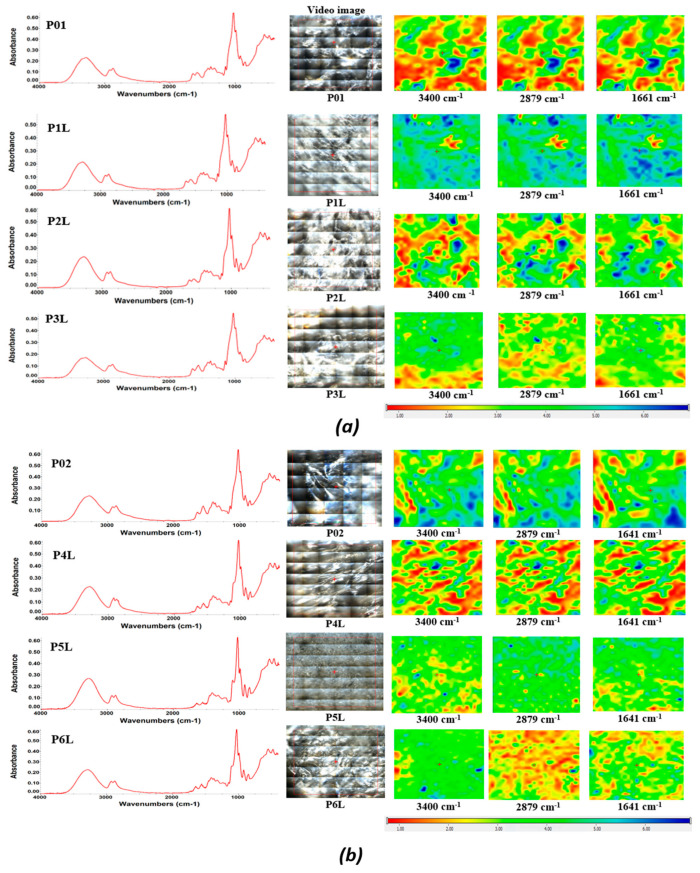
The FTIR maps for the composite chitosan: (**a**) chitosan loaded with lavender essential oil, P01/P1L/P2L/P3L; (**b**) chitosan loaded with AgNPs and lavender essential oil, P02/P4L/P5L/P6L. Red areas indicate the highest absorbance, while blue areas correspond to the lowest absorbance.

The samples’ homogeneity was monitored by FTIR spectroscopy and microscopy, as observed in Figure 1. The FTIR spectra present some characteristic absorption bands. The large band from 3200–3400 cm⁻^1^ is generated by the stretching vibration of the –OH and –NH moieties on the polymeric backbone. The minor split of the band into two peaks at 3335 and 3287 cm⁻^1^ is due to this double nature. The peaks from 2800 to 3000 cm⁻^1^ are from C-H stretching vibrations, are assigned to the methyl and methylene C-H symmetric (υ_CH3_ 2874 cm⁻^1^) and asymmetric (υ_CH2_ 2923 cm⁻^1^) vibrations, and are originating from all organic compounds (chitosan and lavender essential oil). The bands from 1652 and 1559 cm⁻^1^ are assigned to the amide I and amide II vibrations from chitosan. The peak from 1151 cm⁻^1^ is assigned to the asymmetric C-O-C vibration, while the very intense band from 1027 cm^−1^ can be attributed to the glycosidic bond in the polysaccharide chain. In the samples with lavender essential oil, small peaks can be observed in the region of 1700–1750 cm⁻^1^ due to the C=O stretching vibration and the shoulder at ~3080 cm⁻^1^ for the C-H vibrations where carbon is unsaturated (sp or sp^2^).

Considering the specific peaks of the pure LEO and the corresponding wavelengths from the sponges, it can be seen that the components of the LEO interact with the support, and this makes it possible for these samples to maintain the components of the essential oil for a longer period of time. 

Based on the FTIR maps, it can be seen that there is a very good similitude between the maps recorded at the wavelengths specific to chitosan and LEO, at least for a low LEO content. At a higher content of LEO, some small differences appear, which means a slightly preferential adsorption of the components of the essential oils in the sponge, although the homogeneity is still good even at the highest content.

### 2.2. SEM

The morphology of the samples based on chitosan prepared within this study was performed with SEM analyses. The micrographs presented in the next figures exhibit some pictures of the surfaces and cross sections of samples. 

**Figure 2:**
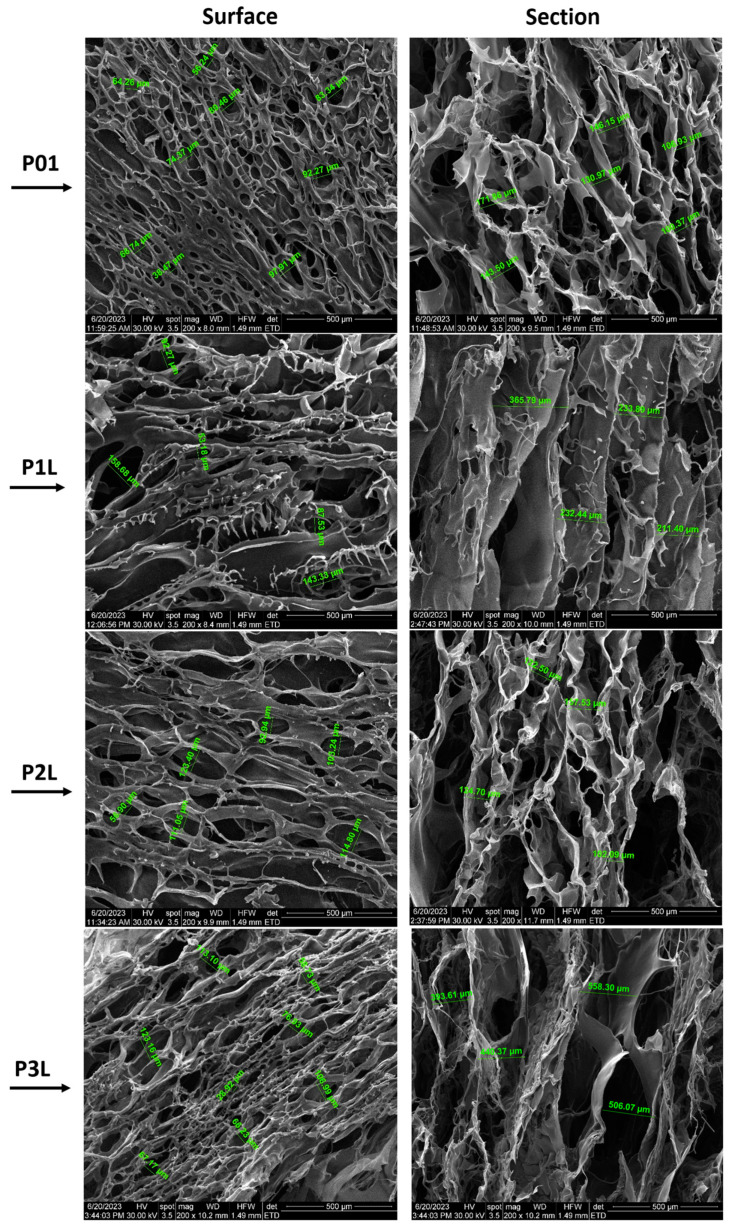
SEM micrographs of P01–P3L samples (200× magnification): surface and section of samples.

**Figure 3:**
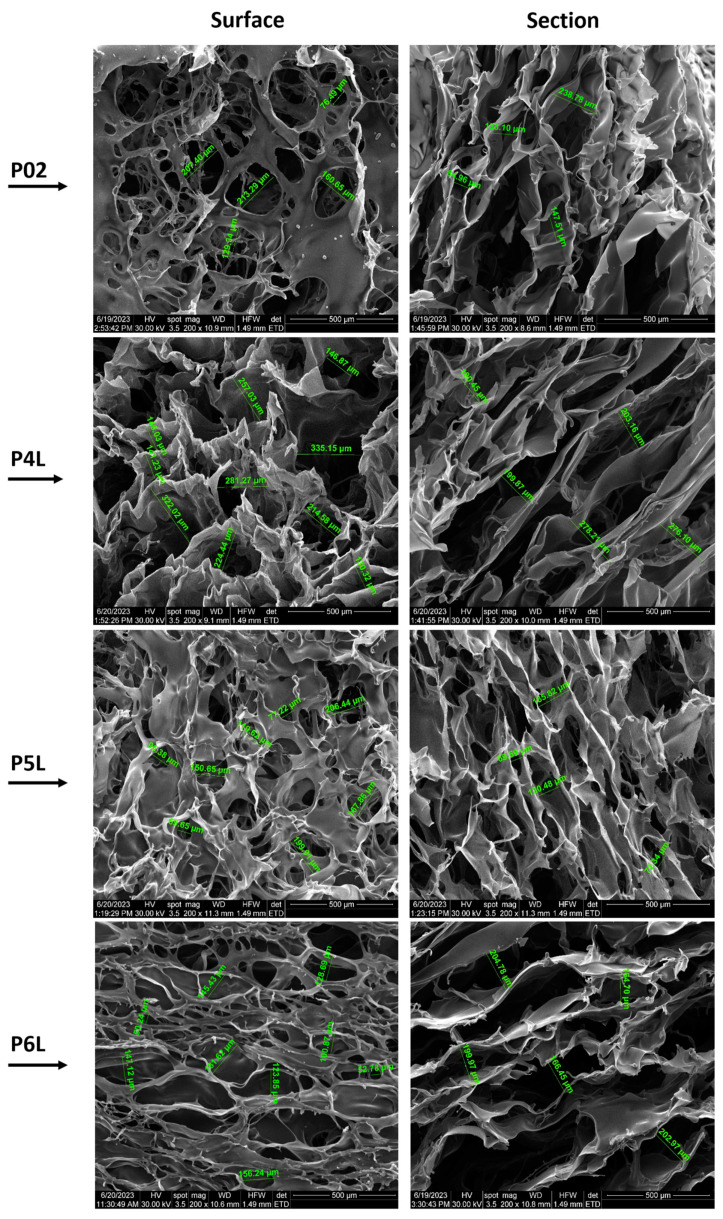
SEM micrographs of P02–P6L samples (200× magnification): surface and section of samples.

Figure 2 presents SEM micrographs of samples P01–P3L, both on the surface and on the section of samples with 200× magnification, while Figure 3 presents SEM micrographs of samples P02–P6L, both on the surface and on the section of samples with 200× magnification. SEM micrographs with 100× magnification of samples P01–P3L and P02–P6L both on the surface and on the section of samples are presented in Figure S1, respectively Figure S2.

SEM micrographs of the P01 surface present a network of open pores with different diameters (38.47–97.91 µm) and different thicknesses of the walls. The high density of the pores and large spectrum of the pore diameters can be observed on the surface of the sample. The cross section of the sample presents a structure with interconnected macropores (106.93–171.86 µm) with an almost uniform long channel shape along the entire cross section of the sample. 

The P1L sample presents a lower pore density, larger diameters (53.18–158.68 µm), and pores that are crowded into each other. The transversal section reveals an organized structure of interconnected pores. The influence of lavender oil can be observed in the mode of the cross-linking of chitosan and the creation of micropores. The cross-section microimage of the sample shows fusiform pores with longer lengths (211.40–365.79 µm) than the ones present in sample P01, which cross the sample material on the entire cross section. Also, a kind of layering of the pores, thinner walls of the pores and some agglomerations of material can be observed.

The surface morphology of the P2L sample shows that the distribution of pores is parallel to their length, with elongated shapes and diameters ranging between 56.9 µm and 123.4 µm. The pore channels are longer oriented along the surface. In the cross section, the uneven distribution and density of pores can be observed. Here, the content of 3% lavender essential oil leads to a more extensive pore network.

SEM micrographs of the P3L surface show a multitude of voids that represent the outer end of the pores with the dimension of the diameters between 28.92 µm and 123.16 µm. In the cross section, an extensive network of interconnected pores can be seen: those with a length that crosses the entire material. The sample shows both large diameters (506.08–558.3 µm) and pore wall thicknesses greater than those of samples P01, P1L, and P2L.

The surface morphology of the P02 control sample containing AgNPs shows a discontinuous film that covers most of the voids, with diameters ranging between 76.49 µm and 213.29 µm. In the cross section, the uneven density and dimensions of pores (84.96–238.78 µm) can be observed.

The micrographs of the P4L sample surface show well-defined pores with diameters ranging between 130.32 µm and 335.15 µm. In the cross section, a lacy form of pores with a more fusiform shape can be observed. The pore density is more compact for pores with smaller diameters, among which filiform pores with much larger diameters are also distinguished. The distance between the pore walls is relatively uniform, with values between 180.45 µm and 278.21 µm.

Compared to sample P4L, the surface of sample P5L is furrowed by a multitude of pores with circular and fusiform shapes and different sizes (77.22–206.44 µm). In the cross section, a homogeneous material without agglomerations or inclusions can be observed, with an extensive, non-uniform porous density and pore dimensions (69.59–180.48 µm).

Unlike samples P4L and P5L, the surface of sample P6L is pierced by a multitude of pores with circular and fusiform shapes and different sizes (50.24–181.62 µm). In the cross section, a uniform structure with an extensive network of pores with interconnected fusiform shapes is observed. The sample material is homogeneous, without agglomerations or inclusions and pore dimensions (164.7–204.78 µm).

### 2.3. Swelling Ratio and Degradation Rate in PBS Solution 

The behavior of samples in contact with PBS was studied by evaluating the swelling degree and mass loss (ISO 62 and ASTM D 570) [87,88]. The solubility in PBS of the analyzed samples represents a characteristic of biodegradability, which offers the potential benefits of applicability in the absorption of biological fluids in the case of surgical interventions or accidents. To evaluate the sample stability, the samples were subjected to swelling measurements to determine the weight change during PBS immersion. 

During the experiment of immersing the samples in PBS, we can distinguish three stages:

- Stage I (0–2 h): PBS penetrates the pores of the material, and the value of the mass of the samples registers a dynamic increase;
- Stage II (2–4 h): the phenomenon of absorption of PBS in the mass of the material and swelling takes place, during which the dimensions of the samples and, implicitly, the mass increase until liquid saturation occurs;
- Stage III (4–8 h): the mass of the samples stabilizes and keeps an approximately constant value.

Stages II and III, in which PBS is absorbed into the sample material, correspond to the beginning and progression of the degradation process through the mass loss in the liquid environment. Specifically, the soluble components of the polymer compounds diffuse in PBS, leaving only the solid structure obtained by cross-linking chitosan with glutaraldehyde.

**Figure 4:**
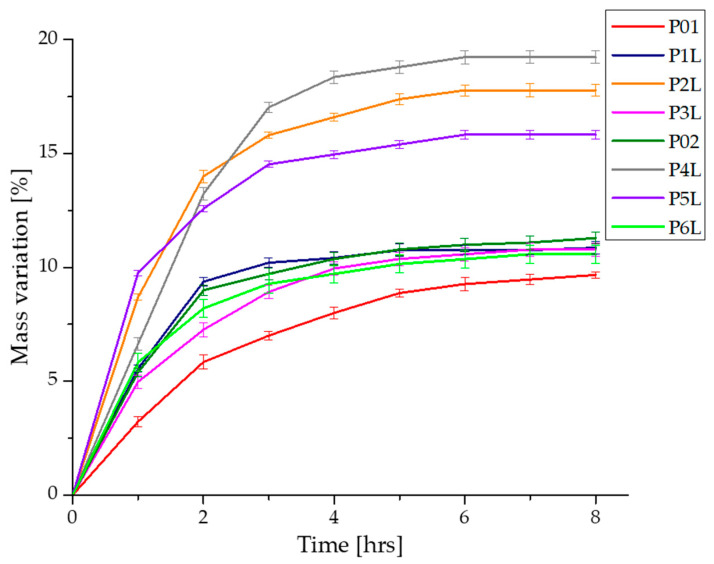
Variation in the mass samples during immersion in PBS.

To evaluate the absorption profile, the value of their masses recorded every hour is represented in Figure 4. The increase in the mass of the samples is due to the affinity of the polymeric composition to the PBS fluid. In the first 2 h, a spectacular mass increase is estimated, corresponding to the filling of pores with PBS and the penetration of fluid into the polymer mass. For up to 4 h, a considerable increase in mass is recorded, after which a fairly low variation in the sample masses and stabilization on an almost constant plateau are observed for up to 8 h. In the case of samples P02–P6L, an improvement in the absorption capacity of PBS in chitosan compositions is observed, due to the presence of silver nanoparticles. A more dynamic absorption profile is registered in the first 3 h, after which the increase in the value of the sample masses is gradually made until the values recorded after approximately 5 h. Compared to the control samples, P01 and P02, the samples with lavender essential oil content show an increase in PBS absorption and improved swelling, and the P4L, P5L, P6L samples show an increase in PBS absorption superior to P1L, P2L, P3L. In conclusion, the presence of antimicrobial agents, silver nanoparticles, and lavender essential oil in the chitosan compositions improve the absorption of biological fluids, while decreasing the contact time with the affected areas.

**Figure 5:**
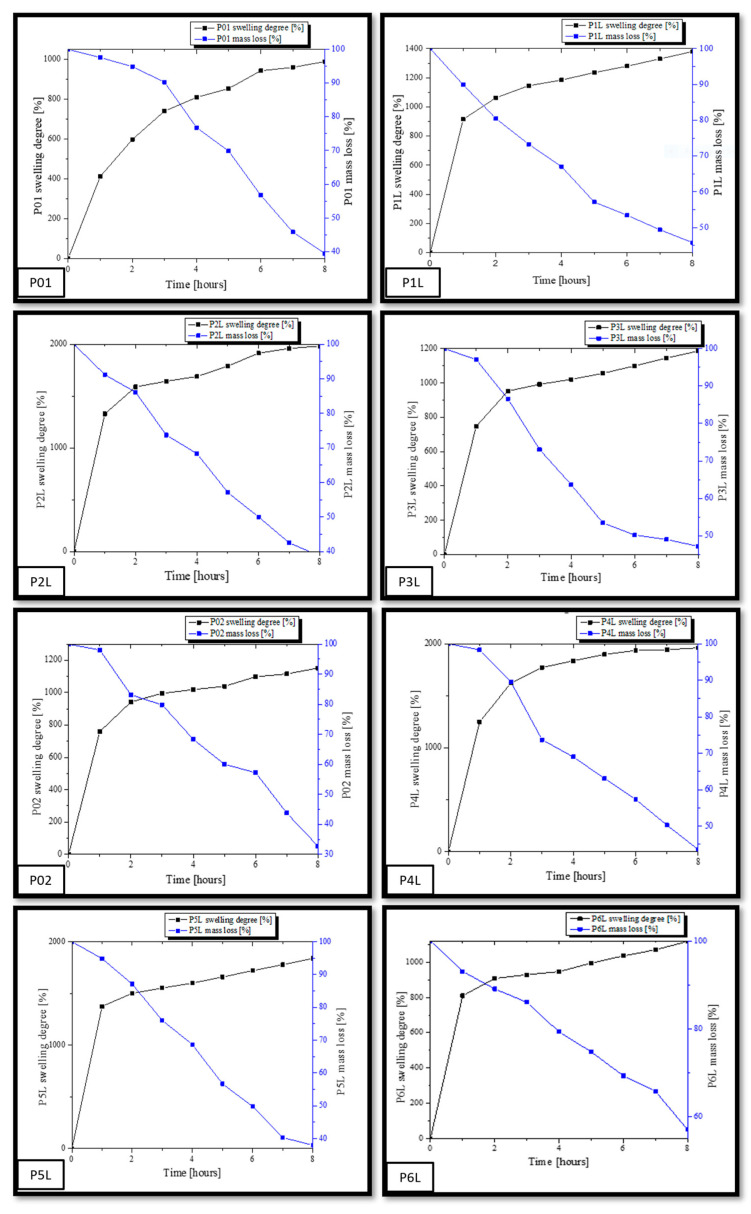
Graphic representation of the swelling degree (SD) variation simultaneously with the mass loss.

Figure 5 shows the profiles of swelling graphs simultaneously with the mass loss during the experiment of immersing the samples in PBS. As previously presented, in the first 4 h, the PBS absorption and swelling are more dynamic, after which the phenomenon stabilizes for each sample. The mass loss of each sample takes place after the absorption of a necessary amount of PBS in the matrix of the chitosan-based composition. At the same time as the absorption of PBS in the polymer matrices, their degradation begins through the mass loss due to the solubility of the components in aqueous fluids.

The swelling capacity of samples P01–P6L was performed by immersing in PBS (pH = 7.4). As mentioned before, we analyzed two categories of samples, namely, a category composed of chitosan and lavender essential oil and a second category composed of chitosan, AgNPs, and lavender essential oil. The immersion experiments in PBS lasted 8 h. The samples were initially weighed at intervals of one hour, until the absorption of PBS reached saturation. The capacity of swelling for each sample was calculated using Equation (1). At the same time, the mass for each sample was registered. From the analysis of the numerical data, it is observed that all samples in the first two hours were dominated by a dynamic process of absorption PBS, after which the swelling evolution was slower until it was found to be inflicted on a balance. The values of the swelling capacity and the mass loss for each sample are different depending on their composition. Thus, a swelling capacity for the control sample, P01, of about 989.11% (the small value between all samples) and a final mass loss of 39.46% are observed. This is due to the smaller dimensions of the pore channels in the mass of the witness sample, as observed in the SEM micrographs. In contrast, the P1L, P2L, and P3L samples containing lavender essential oil registered higher swelling capacities by 1380.56%, 1983.4%, and 1185.48%. It is worth noting that the sample, P2L, with a content of 3% lavender essential oil presented the highest swelling capacity in this category. 

In the case of the samples with content of AgNPs and essential oil, P4L, P5L, and P6L, the same trend in the capacity of swelling is noted. Thus, the sample with the highest content of the essential oil of lavender, P6L, presented a lower increase in the capacity of swelling (1118.36%) compared to the sample control, P02 (1150%). In contrast, the P4L sample with 1% lavender essential oil presented the highest increase in the capacity of swelling compared to the sample control, P02, namely, 1960.62%. The P5L sample presents a value of the swelling capacity close to that of the P4L sample, namely, 1838.83%. These values are in accordance with the interactions developed between AgNPs and the essential oil of lavender within the chitosan matrix. There is a large absorption capacity of PBS in the compositions based on chitosan and 3% lavender essential oil, and, if added, the swelling capacity reaches an acceptable value for 1% and 3% lavender essential oil. Similar research performed on chitosan compositions showed different values of swelling capacity, for example, more than 2300% [32], 1000% [89], and 300% [90].

At the same time, it can be seen that there is a larger mass loss in the case of samples with a content of 5% lavender essential oil compared to the studied samples. Analyzing the mass loss, we can conclude that increasing the amount of lavender essential oil favors the degradation of samples by the dissolution, fragmentation, and diffusion in PBS of a larger amount of the components from the compositions based on chitosan made within this work. As a conclusion, we can assert that a content of 1% and 3% lavender essential oil in chitosan porous compositions can be useful in medical applications for absorbing biological fluids, while a content of 5% essential oil is effective for applications that require the biodegradation of devices in contact with open lesions for the release of quantitative components.

### 2.4. UV–Vis and Fluorescence (PL) Spectroscopy

**Figure 6:**
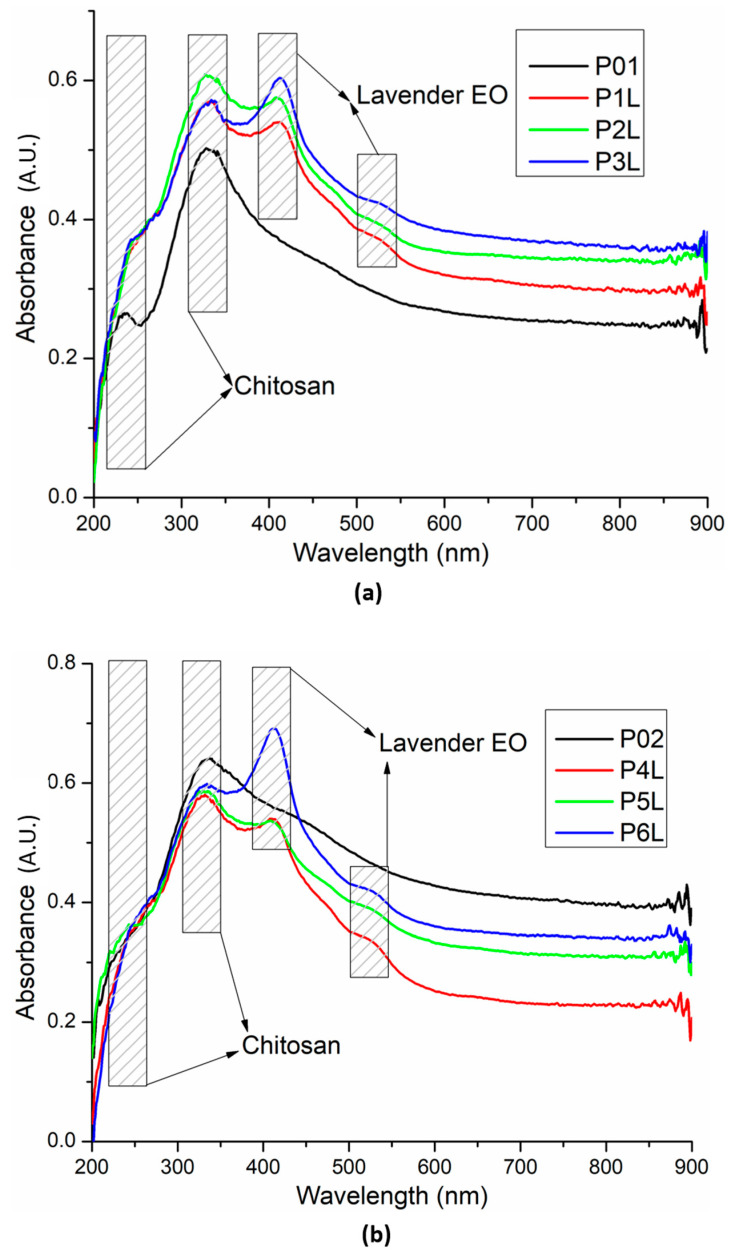
UV–Vis spectra for the two chitosan-based series: chitosan and lavender essential oil (**a**) and chitosan, Ag NPs, and lavender essential oil (**b**).

The UV–Vis spectra for the two series of chitosan-based sponges are comparatively presented in Figure 6. The presence of Ag NPs in the chitosan matrix is generating subtle modification due to the new interactions generated with moieties like –OH. The band at 235 nm is increasing in intensity, becoming a shoulder of the more intense peak at 330 nm. Additionally, the samples have a slightly higher absorbance in the visible domain. The well-known peak of Ag NPs from around 400 nm, due to localized surface plasmon resonance, is generally masked by the peaks of lavender essential oil and the shoulder of the chitosan’s large absorption band, which is responsible for the yellowish tint of the sponges [84].

**Figure 7:**
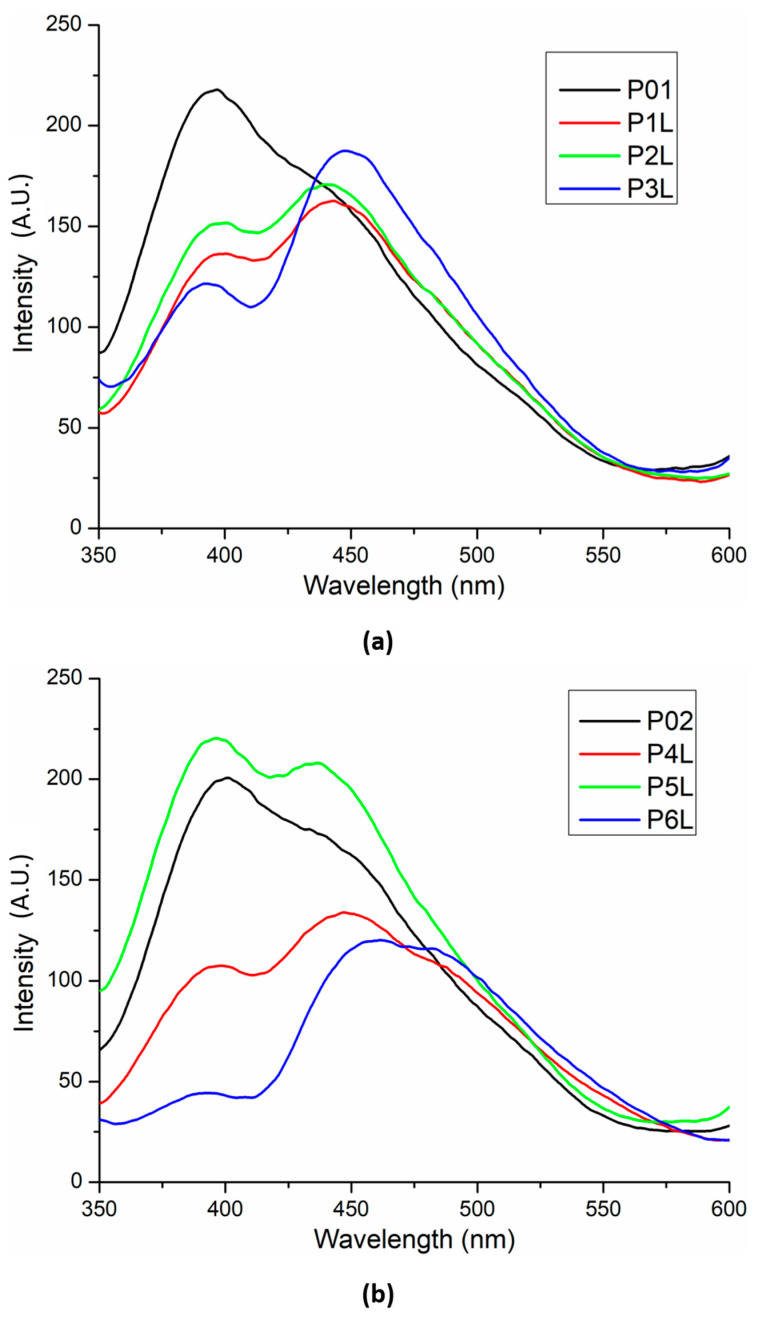
Fluorescence spectra for the two chitosan-based series: chitosan and lavender essential oil (**a**) and chitosan, Ag NPs, and lavender essential oil (**b**).

Fluorescence spectra for the two chitosan-based series are presented in Figure 7. The introduction of Ag NPs in the chitosan matrix slightly modifies (decreases) the intensity of the chitosan’s UV fluorescent emission (~395 nm) but, at the same time, marginally increases the intensity of the visible emission (shoulder from ~450 nm). In addition, the whole emission spectrum suffers a bathochromic shift of ~5 nm.

The presence of lavender essential oil generates the split of the large emission band into two distinct bands, in general with a lower intensity due to quenching interactions between chitosan and the components of lavender essential oil. 

### 2.5. Complex Thermal Analysis: TG-DSC-FTIR

**Figure 8:**
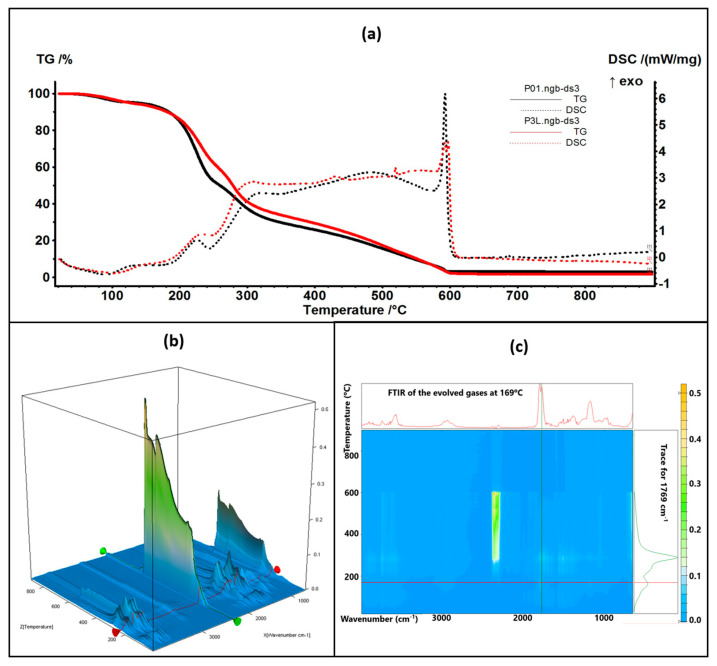
The TG-DSC curves for the P01 and P3L samples (**a**); FTIR 3D diagram of the evolved gases (**b**) and its 2D projection in the wavenumber/temperature plan for the P3L sample (**c**)—upper side is the FTIR spectrum at 169 °C, and right side is the trace for C=O (1769 cm^−1^).

**Figure 9:**
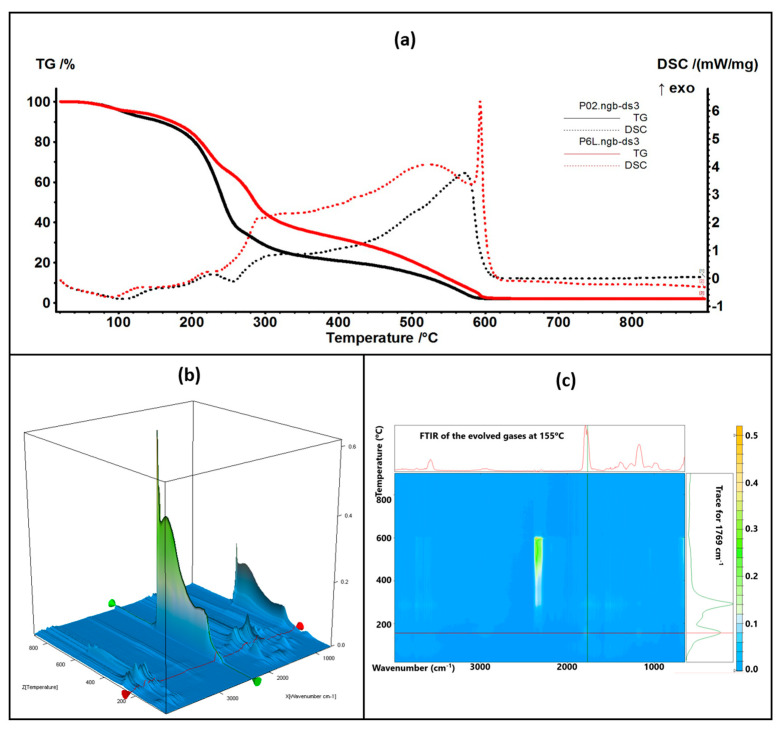
The TG-DSC curves for the P02 and P6L samples (**a**); FTIR 3D diagram of the evolved gases (**b**) and its 2D projection in the wavenumber/temperature plan for the P6L sample (**c**)—upper side is the FTIR spectrum at 155 °C, and right side is the trace for C=O (1769 cm^−1^).

The thermal analysis results are comparatively presented between the control samples (simple chitosan or chitosan with Ag NPs) and the corresponding sample containing 5% lavender essential oil (Figure 8 and Figure 9).

The thermal analysis curves of TG and DSC are similar for the control and containing essential oil samples. The numerical values are presented in Table 1. The P3L sample loses 6.41% of its initial mass up to 150 °C, and the process is accompanied by an endothermic effect, with a minimum at 90 °C. This indicates that the most probable cause is the elimination of some residual water molecules as well as the volatile components from lavender essential oil. The FTIR spectra of the evolved gases indicate the presence of water molecules at 90 °C and some traces of organic compounds. Between 150 and 255 °C, the sample loses 33.22% of its mass in a complex degradative process, which is accompanied by endothermic and exothermic effects on the DSC curve. The FTIR spectra of evolved gases indicate that at 169 °C CO_2_ and H_2_O are present, suggesting the partial oxidation of chitosan as well as organic fragments that are present (FTIR bands corresponding to C-H stretching vibrations in the interval of 2800–3000 cm^−1^ and the C=O vibration from 1769 cm^−1^, indicating the decomposition of organic compounds) [84]. As temperature increases, C-H-specific symmetric and asymmetric vibrations are observable along CO-specific vibrations at 225 °C as minor components in the evolved gases, with the bulk consisting of CO_2_ and H_2_O, indicating the predominance of oxidation over decomposition. Strong oxidation continues up to 620 °C, when residual carbonaceous mass is burned away, and the recorded mass loss is 58.36%.

The P6L sample exhibits a mass loss of 7.01% up to 150 °C via similar processes as P3L. The complex process (oxidation and decomposition) between 150 and 255 °C is responsible for a mass loss of 29.13%. For both cases, P3L and P6L, the addition of lavender essential oil leads to a slower decomposition rate after 200 °C, when compared with the control samples, probably due to the partial cross-linking of chitosan polymer chains by functional compounds from the essential oil.

### 2.6. Antibacterial Analysis

#### 2.6.1. Qualitative Screening for Evaluation of the Antibacterial Efficiency of Lavender Essential Oil

**Figure 10:**
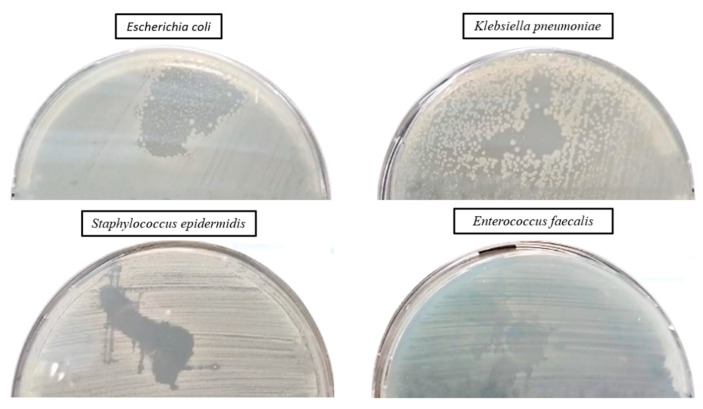
Aspect of the inhibition zone of lavender essential oil used for samples’ preparation.

The qualitative assessment of the antibacterial activity of lavender essential oil was conducted using an adapted spot diffusion method, following the CLSI standard for 2022 [91]. This procedure involved applying 20 µL of lavender essential oil onto the surface of agar medium inoculated with microbial cultures at a standard density of 1.5 × 108 CFU/mL, with the goal of determining the diameter of the inhibition zone. An aspect of the inhibition zone of the lavender essential oil used for the samples’ preparation is shown in Figure 10. During incubation, two drops were dispersed on the surface of the medium and merged, generating the irregular common inhibition zone for which the exact measurement of the diameter was not possible (Figure 10). The effectiveness of the antimicrobial activity was subsequently quantitatively evaluated, with the MIC values (%) being added to Table 2.

#### 2.6.2. Qualitative Results Regarding the Antibacterial Efficiency of Tested Samples

**Figure 11:**
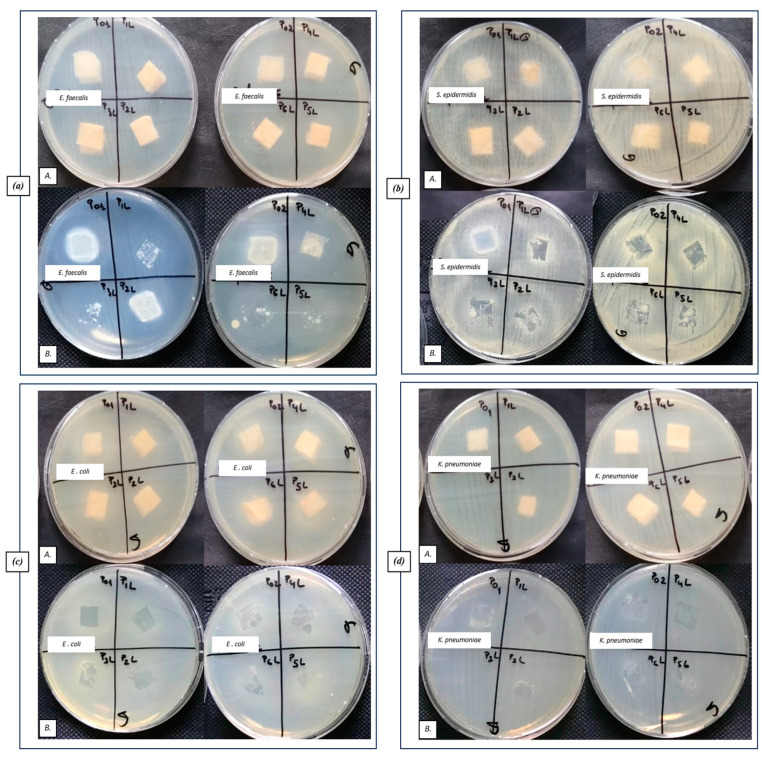
Aspect of the bacterial cultures, after placing the samples on the surface of the culture medium (**A**) and after removing the samples (**B**): (**a**) Gram-positive *E. faecalis* VRE, (**b**) Gram-positive *S. epidermidis* 2018, (**c**) Gram-negative *E. coli* ATCC 25922, and (**d**) Gram-negative *K. pneumoniae* ATCC 10031.

Qualitative results, regarding the antibacterial activity of different biomaterials, were obtained after their contact with the sensitive strains dispersed on the agar medium, as observed in Figure 11.

No inhibition zone extended over the contact area can be observed for most tested samples (Figure 11(aA,cA,dA)). An inhibition zone can be observed only on the contact area of the tested sponge samples with the medium (Figure 11(aB,cB,dB)), especially for the P1L (CH + 1% LEO) and P4L (CH + AgNP + 1% LEO) samples.

#### 2.6.3. Quantitative Evaluation of the Antibacterial Efficiency of Functionalized Samples

**Figure 12:**
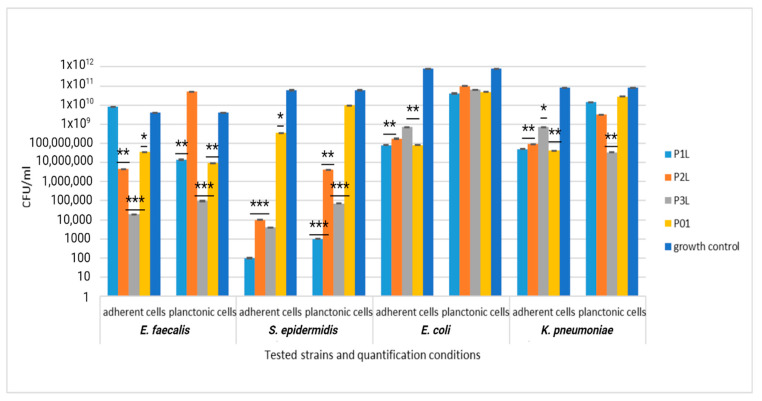
Graphic representation of CFU/mL for comparative evaluation of the antibacterial activity of tested sponge samples loaded with lavender essential oil. The results were compared using two-way ANOVA and Dunnett’s multiple comparisons tests; ns—not significant; * *p* < 0.05; ** *p* < 0.001; *** *p* = 0.0001.

A quantitative evaluation of the antibacterial efficiency of functionalized samples was assessed using the VCC method. When the sponge samples were loaded only with lavender essential oil, the inhibitory effect was significantly manifested only in the case of Gram-positive strains, especially against *S. epidermidis*. Thus, both the multiplication of planktonic cells and their adhesion to the biomaterial were significantly inhibited, compared with the growth control (represented by the corresponding microbial strain cultivated in the broth medium) and sponge sample control (P01), as observed in Figure 12.

For Gram-negative strains *E. coli* and *K. pneumoniae*, the inhibitory effect was significantly manifested only against the cells adhering to the biomaterial, which was less obvious against the planktonic cells, as observed in Figure 12.

**Figure 13:**
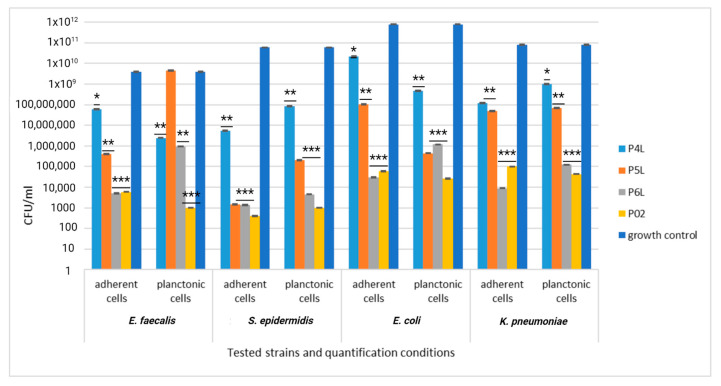
Graphic representation of CFU/mL for comparative evaluation of the antibacterial activity of tested sponge samples loaded with AgNPs and lavender essential oil. The results were compared using two-way ANOVA and Dunnett’s multiple comparisons tests; ns—not significant; * *p* < 0.05; ** *p* < 0.001; *** *p* = 0.0001.

The addition of AgNPs significantly improves the efficiency of the sponge sample control (P02), exhibiting a significant inhibitory effect against all tested bacterial strains (yellow column, Figure 13). The combination of lavender essential oil at different concentrations does not potentiate the inhibitory effect either against the cells adhering to the sponge sample or against the planktonic cells; the CFU/mL values are even higher, in some cases, compared to the values recorded for the control sample.

## 3. Materials and Methods

### 3.1. Materials

#### 3.1.1. Raw Materials

Chitosan powder (with a low molecular weight), glycerol, absolute ethanol, and glutaraldehyde (GA) (50% in water) were purchased from Sigma-Aldrich (Saint Louis, MO, USA). Phosphate Buffer Solution (PBS) was purchased from Sigma Aldrich (Saint Louis, MO, USA with pH-7.4 according to product technical sheet). Glacial acetic acid (AcA) was purchased from Chimreactiv (Bucharest, Romania), and double-distilled water was used. Lavender essential oil (LEO) was purchased from S.C. HOFIGAL S.A. Bucharest (Bucharest, Romania).

Culture medium, Muller Hinton for bacteria strains, and Sabouraud for yeast strain were supplied by Scharlau Group (Scharlau 02-136-500, Barcelona, Spain). HT-29 cells were grown in RPMI 1640 (Lonza, Switzerland) and were supplemented with 10% FBS (fetal bovine serum) and penstrep (Biochrom, Germany). MTT reagent was purchased from Sigma Aldrich (Saint Louis, MO, USA).

The antimicrobial assay was performed using standard strains, from the collection of University of Bucharest, Microbiology Department: *Staphylococcus epidermidis* 2018 and *Enterococcus faecalis* VRE 2566 (Gram-positive strains) and *Klebsiella pneumoniae* ATCC 10031 and *Escherichia coli* ATCC 35218 (Gram-negative strains).

AgNPs were synthetized as previously described [92]. Briefly, 0.02 g AgNO_3_ was dissolved in 100 mL water under vigorous stirring at 70 °C. Then, 20 mL solution of 0.35 g sodium citrate was added dropwise as a reduction agent. After 30 min, a third solution (0.1 g PVP in 5 mL) was added dropwise. The yellow solution containing AgNPs (100 ppm) was used without further purifications.

#### 3.1.2. Preparation of the Chitosan Sponges 

Chitosan solution was formed by dissolving chitosan powder in 100 mL of 3% acetic acid glacial solution (AcA). The prepared solution was stirred for 24 h at room temperature. Afterward, 1 mL glycerol was added, and the solution was stirred for another 2 h at room temperature. 

Another chitosan solution was prepared as previously described, in which, under vigorous stirring, silver nanoparticles (AgNPs) suspension was added.

The polymeric solutions formed were cast on Petri dishes and then lyophilized in a freeze dryer. 

A solution of glutaraldehyde (5%wt) mixed with 150 mL absolute ethanol was prepared for cross-linking the lyophilized polymer. Then, variable volumes (1 mL, 2 mL, and 5 mL) of lavender essential oil (LEO) were added to the glutaraldehyde and absolute ethanol solution. These solutions, formed with lavender essential oil (LEO) in different concentrations, were sonicated for 30 min.

Essential oil concentrations were chosen to obtain series of samples with variable content of lavender essential oil (LEO), as presented in Table 3. The samples were dried in an oven for 24 h at 40 °C.

All experiments were carried out in triplicate, allowing for the minimization of impact any potential variability or outliers that could affect the accuracy of our research.

### 3.2. Characterization Methods

#### 3.2.1. FTIR Spectroscopy

Fourier-transform infrared spectra were recorded with a Nicolet iS50 FTIR spectrometer (Thermo Fisher Scientific Inc., Waltham, MA, USA), with an ATR module, in the 4000–400 cm^−^^1^ domain, with a resolution of 2 cm^−^^1^; each spectra was an average of 32 scans.

FTIR 2D maps were recorded with a Nicolet iS10MX FTIR microscope (Thermo Fisher Scientific Inc., Waltham, MA, USA), in the 4000–650 cm^−^^1^ range.

#### 3.2.2. UV–Vis Spectroscopy

A JASCO V560 spectrophotometer (JASCO Inc., Easton, PA, USA) was used to measure the UV–Vis spectra. The device was equipped with a 60 mm integrating sphere (ISV-469) and a film holder for the samples. The spectra were recorded with a speed of 200 nm min^−1^, in the domain of 200–900 nm.

#### 3.2.3. Fluorescence (PL) Spectroscopy

A Perkin Elmer (Waltham, MA, USA) LS55 spectrometer was used to measure the photoluminescence spectrum (PL). A Xe lamp was used as a UV light source at ambient temperature, with the fluorescence being measured in the range of 350–800 nm. The spectra were recorded with a scan speed of 200 nm min^−1^, excitation and emission slits of 10 nm, and a 350 nm cut-off filter. An excitation wavelength of 320 nm was used.

#### 3.2.4. SEM

The surface morphology and microstructure of the experimental samples were evaluated with a FEI QUANTA INSPECT F (FEI Company, Eindhoven, The Netherlands) scanning electron microscope, providing a resolution of 132 eV at MnKα. The examination of microstructural features was conducted subsequent to gold coating the sample’s surface in an argon atmosphere, under high-vacuum operational conditions. We obtained images at 200× magnification and 100× magnification (Supplementary Materials). 

### 3.3. Evaluation of the Samples Degradation in PBS

The behavior of sponge samples based on chitosan P01–P6L were studied by immersion of samples in phosphate-buffered saline (PBS) solution, with pH of 7.4, as a simulating fluid.

Samples were cut in parallelepipedal shape (10 mm × 10 mm × 5 mm). Before testing, the samples were dried in a desiccator to achieve constant weight. All experiments were carried out in triplicate, and the mean value was used to create the graphs.

#### 3.3.1. Swelling Properties

Sponge samples were immersed in PBS solution at room temperature (27 °C). In contact with PBS, because of porous structure, samples show intense fluid absorption. This aspect determined an increase in volume simultaneously with the expansion of the 3D dimensions and implicitly of the mass of the studied samples. During the immersion experiment, the samples were weighed at intervals of one hour, until the recorded masses were constant (approximately 8 h). The swelling degree (SD) of samples was calculated with Equation (1), taking into account the mass of each sample both at the beginning of the experiment, *m_i_* [g], and at different time intervals until the end of the experiment, *m_s_* [g] [84,93].

(1) $$SD=\frac{m_{s}-m_{i}}{m_{i}}\times 100$$

#### 3.3.2. Mass Loss

The immersion test of the experimental samples was carried out in 40 mL of PBS with a pH value of 7.4 at 37 ± 0.5 °C. The test evaluates the degradation behavior through mass loss. During the test, the PBS solution was changed every day at the same hour. During the experiment, the medium easily penetrates inside the polymer material, with the diffusion being favored by the thinness of the pore walls. Degradation of samples in PBS was calculated with Equation (2).

The samples were weighed at the beginning, m_i_, and after every hour during the experiment. After 8 h, the samples were weighed for final evaluation, *m_f_* [94,95].

(2) $$Mass\;loss=\frac{m_{i}-m_{f}}{m_{i}}\times 100$$

where *m_i_* is the initial mass value recorded at the beginning of the experiment, and *m_f_* is the final mass value at the end of the experiment.

### 3.4. Thermal Analisys

Thermal behavior was followed with a STA 449C F3 system, TG-DSC (thermogravimetry–differential scanning calorimetry) from Netzsch (NETZSCH-Gerätebau GmbH, Selb, Germany), between 20 and 900 °C, in dynamic (50 mL/min) air atmosphere. The evolved gases were transferred through heated transfer lines and analyzed on the fly with the help of a FTIR Tensor 27 from Bruker (Bruker Co., Ettlingen, Germany), equipped with an internal thermostatic gas cell.

### 3.5. Antibacterial Analysis

#### 3.5.1. Qualitative Method for Evaluation of the Antibacterial Efficiency of Lavender Essential Oil

For the qualitative screening of the antibacterial activity, an adapted spot diffusion method was followed (according to CLSI standard 2022) [91] in order to determine the diameters of the inhibition zone expressed by the lavender essential oil. An amount of 20 µL was spotted on the surface of the agar medium seeded with bacterial inoculums at standard density of 1.5 × 10^8^ CFU/mL. The antibacterial assay was performed using standard strains, from the collection of University of Bucharest, Microbiology Department: *Staphylococcus epidermidis* 2018 and *Enterococcus faecalis* VRE 2566 (Gram-positive strains) and *Klebsiella pneumoniae* ATCC 10031 and *Escherichia coli* ATCC 35218 (Gram-negative strains).

#### 3.5.2. Qualitative Method for Evaluation of the Antibacterial Efficiency of Tested Samples

Standard inoculum, represented by bacterial cell suspensions with 1.5 × 10^8^ CFU/mL density, was uniformly distributed on the surface of the agar medium. Subsequently, fragments of functionalized sponge samples, with diameter of 1 cm, were placed on the surface of the medium and lightly pressed to come into direct contact with the medium. After the free diffusion of the active components, the plates were incubated for 16–18 h at 37 °C. The antibacterial effect was assessed according to the inhibition zones expressed in the contact zone with the culture medium and outside this zone.

#### 3.5.3. Quantitative Method for Evaluating the Antibacterial Efficiency of Functionalized Sponge Samples by Determining CFU/mL Values

The quantitative evaluation of the antibacterial efficiency of the functionalized sponge samples was carried out both by determining the CFU/mL values for the bacterial cells adhered to the surface of the functionalized sponges (adhered cells) and by determining the CFU/mL values for the planktonic cells. For this purpose, sterile plates with 24 wells were used; 1 mL of liquid culture medium was added to each well, which was inoculated with 10 µL of standardized bacterial suspension, followed by immersion of the tested samples. The plates were incubated at 37 °C, for 24 h, during which the microbial cells initially multiplied in suspension, and, after reaching a threshold density, they began to adhere to the surface of the materials. After 24 h, each piece of material was removed from the medium, gently washed 3 times with TFS (sterile phosphate buffer) to remove non-adherent cells, and placed in sterile centrifuge tubes containing 1 mL of TFS. The samples were vigorously mixed in the shaker for 1 min and sonicated for 10 s. The quantitative evaluation of cell density (both adhered cells and planktonic cells) was assessed using viable cell count (VCC) method [96].

## 4. Conclusions

Uncontrolled bleeding resulting from a traumatic injury can lead to various complications, significantly increasing healthcare costs and posing a life-threatening risk to the patient. Consequently, it is imperative to employ an efficient hemostatic agent to effectively achieve hemostasis, thereby reducing the likelihood of complications and the associated morbidity and mortality linked to a hemorrhage. Therefore, the development of hemostatic materials with inherent antibacterial properties is crucial for treating wounded individuals and enhancing survival rates. Hence, we developed chitosan-based absorbent sponges incorporating antimicrobial agents like silver nanoparticles (AgNPs) and lavender essential oil (LEO).

The objective of this research was to introduce a novel chitosan sponge, with hemostatic application, equipped with an antibacterial shield effective against a broad spectrum of both Gram-positive and Gram-negative microorganisms. In the research we developed, we used three compositions based on chitosan and lavender essential oil, called P1L, P2L, and P3L, and the control sample was P01. We also made a control sample, P02, composed of chitosan and silver nanoparticles, as well as three samples, P4L, P5L, and P6L, composed of chitosan, essential lavender oil, and silver nanoparticles.

Thus, the P2L sample had the highest swelling degree throughout the experiments until the end after 8 h. The presence of lavender essential oil in the compositions with silver nanoparticles did not influence the swelling degree much. Instead, the P6L sample had a more degradable character, with the highest mass loss of 54%, which can be used in applications with the controlled release of some active principles through degradability in damaged areas of the skin and the absorption of components inside the wound.

In the case of the quantitative evaluation of the antibacterial efficiency, it was found that samples P02–P6L with AgNPs content showed an increased efficiency of the inhibitory effect against all tested bacterial strains. The inclusion of lavender essential oil at various concentrations did not enhance the inhibitory effect on the bacteria.

Future directions for this study involve investigating synergistic approaches by combining chitosan sponges with complementary therapeutic modalities, such as advanced drug delivery systems. This exploration can further enhance the sponges’ capabilities in accelerating wound healing and supporting infection control. In addition, preclinical animal testing would help in rigorously evaluating the safety, efficacy, and biological responses of these chitosan-based sponges.

In conclusion, research on chitosan-based sponges with antimicrobial and hemostatic properties holds significant potential for addressing wound care, surgery, and trauma management. These future directions aim to enhance the practical application, efficacy, and sustainability of chitosan-based sponges in the medical field. 

## Figures and Tables

**Table 1:** Principal values of thermal analysis.

Sample	Mass Loss RT-150 °C	Mass Loss 150–255 °C	Mass Loss 255–620 °C	Endo I	Exo I
P01	5.61%	43.26%	47.98%	90.0 °C	225.5 °C
P3L	6.41%	33.22%	58.36%	102.1 °C	233.3 °C
P02	9.21%	50.07%	38.56%	106.5 °C	228.7 °C
P6L	7.01%	29.13%	61.55%	88.9 °C	227.8 °C

**Table 2:** Qualitative results regarding the antibacterial activity of lavender essential oil.

	Lavender Essential Oil
*Bacterial Strains*	Minimal Inhibitory Concentration (%)
*E. faecalis* VRE 2566	5%
*S. epidermidis* 2018	2.5%
*E. coli* ATCC 35218	0.625%
*K. pneumoniae* ATCC 10031	1.25%

**Table 3:** Sample codification and composition.

Sample Code	Chitosan (g in 100 mL of 3% (*v*/*v*) AcA)	Ag NPs (mL of 100 ppm Solution)	LEO (mL)
P01	2 g	-	-
P1L	2 g	-	1
P2L	2 g	-	2
P3L	2 g	-	5
P02	2 g	2	-
P4L	2 g	2	1
P5L	2 g	2	2
P6L	2 g	2	5

AcA—acetic acid; AgNPs—silver nanoparticles; LEO—lavender essential oil.

## Data Availability

The experimental data on the results reported in this manuscript are available upon official request to the corresponding authors.

## Supplementary Materials

The following supporting information can be downloaded at https://www.mdpi.com/article/10.3390/ijms242216312/s1.
