# Supplementary material for: Influence of Lavender Essential Oil on the Physical and Antibacterial Properties of Chitosan Sponge for Hemostatic Applications
Source: Int J Mol Sci. 2023 Nov 14;24(22):16312. doi: 10.3390/ijms242216312 (PMC10671502; doi:10.3390/ijms242216312)
Supplement: Supplementary file 1 [file ijms-24-16312-s001.zip › ijms-2685630-supplementary.pdf]

Supplementary Material

The following is the supplementary data related to this article.

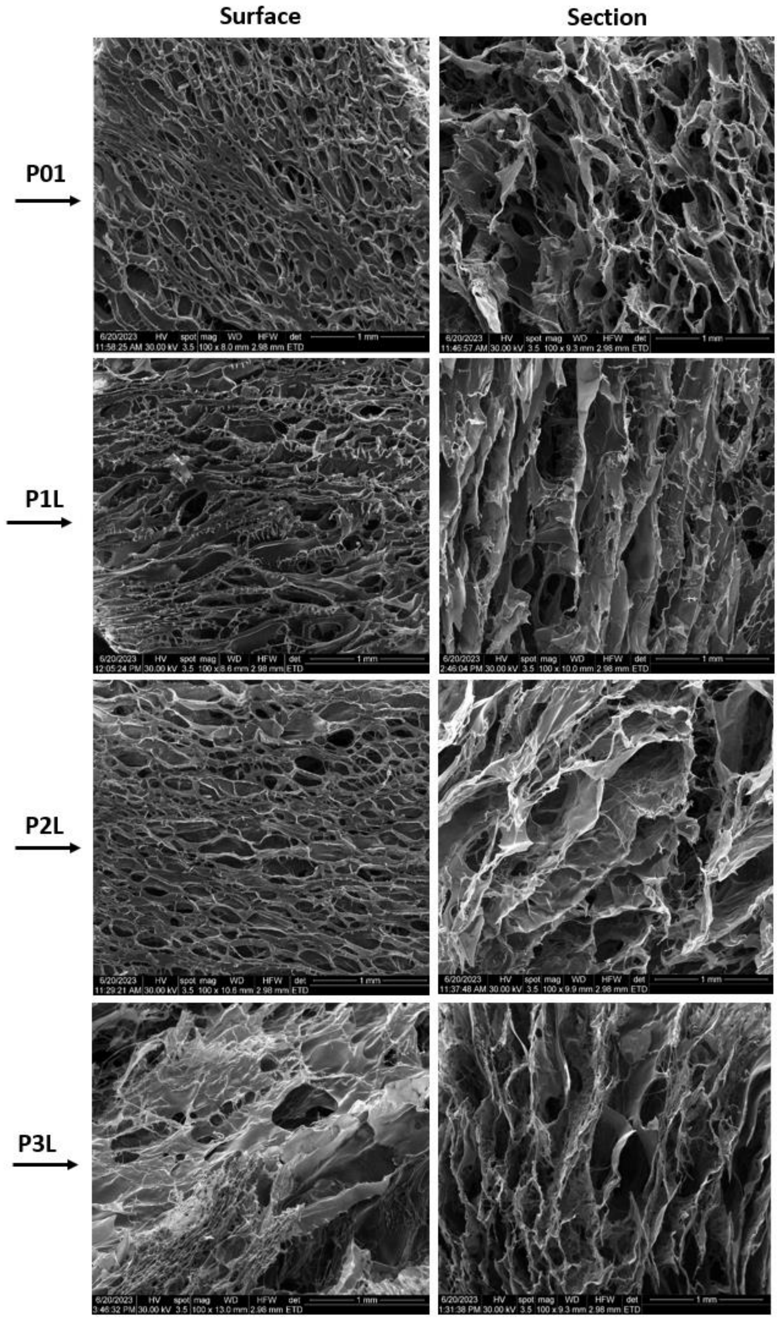

Figure S1. SEM micrographs of samples P01-P3L (100x magnification): surface and section of samples

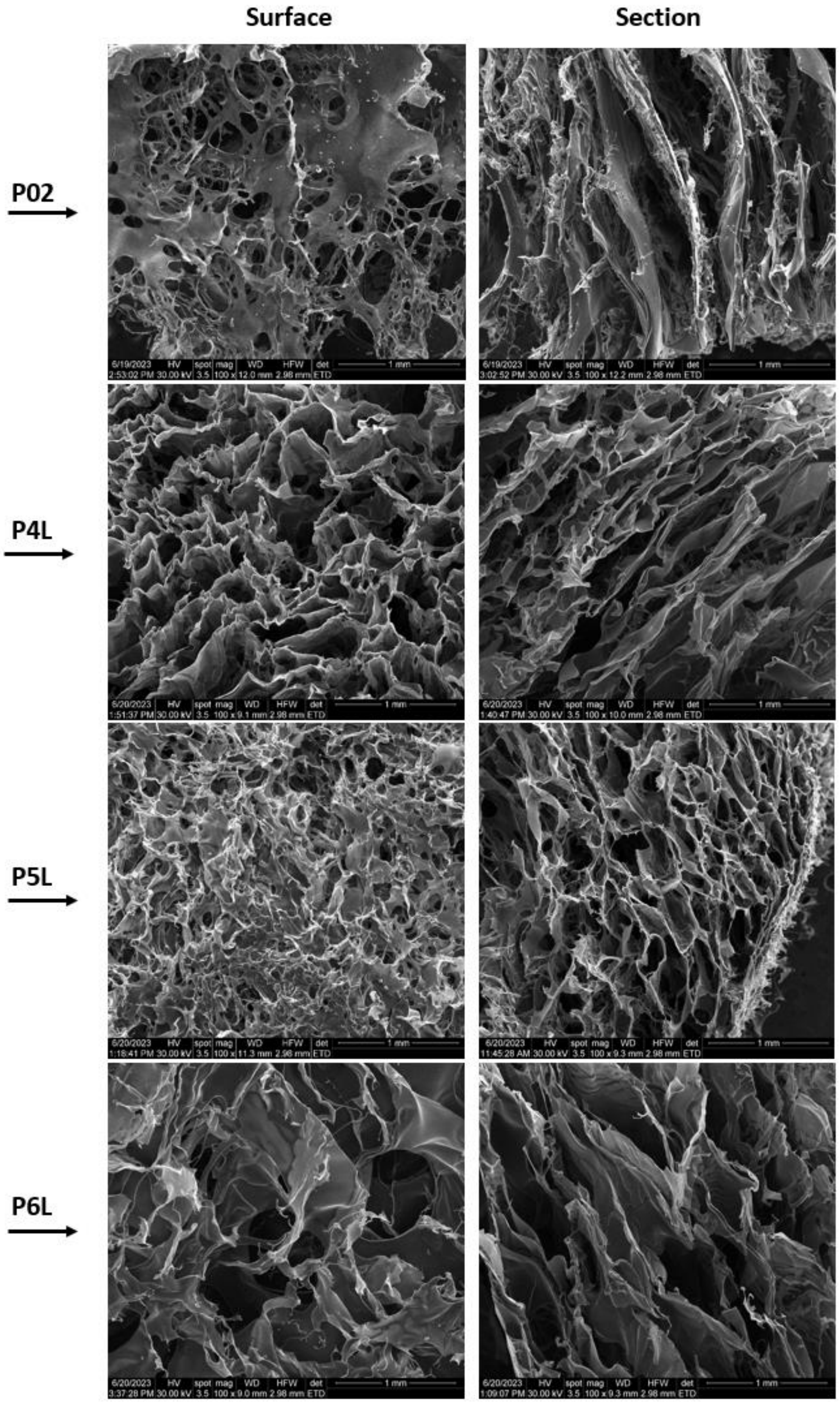

Figure S2. SEM micrographs of samples P02-P6L (100x magnification): surface and section of samples
